# Dual-Task Costs in Gait Speed Differs Across Age Groups

**DOI:** 10.1093/geroni/igab046.3248

**Published:** 2021-12-17

**Authors:** Sally Paulson, Michelle Gray, Joshua Gills, Anthony Campitelli, Megan Jones, Joohee Sanders, Erica Madero, Jordan Glenn

**Affiliations:** 1 University of Arkansas at Fayetteville, Cincinnati, Ohio, United States; 2 University of Arkansas at Fayetteville, Fayetteville, Arkansas, United States; 3 Shippensburg University of PA, Shippensburg, Pennsylvania, United States; 4 Neurotrack Technologies, Redwood City, California, United States

## Abstract

With age, there are simultaneous reductions in gait speed (GS). This decrease in GS has been associated with an increased fall risk and negatively impacts independence. Further, GS naturally declines with the addition of a secondary stimulus (i.e., cognitive requirements). Combined, these decrements can be additive in nature potentially leading to robust declines with advancing age. Therefore, the aim of this study was to examine age-related effects of dual-task cost (DTC) while walking. Adults (N = 145), over the age of 45 years, completed two walking trials for each GS condition: habitual (HAB) and fast (FST), with and without a DT (i.e., counting backwards by serials of three). Subjects were classified into four age groups: youngest-old (YG ≤ 64 years, n = 24), young-old (YO, 65-74 years, n = 46), middle-old (MO = 75-84 years, n = 54), and oldest-old (OO ≥ 85 years, n = 21). DTC was calculated and ANOVAs were used to assess differences between the groups. There was no difference in HAB DTC between the age groups (p=.61). However, there was a significant difference in FST DTC (p=.04) between the YO (M±SD: -14 ± -11%) and OO (M±SD: -24 ± -12%). These data indicate there was an age-related affect for fast dual-task cost, but not for habitual dual-task cost while walking. An increase in dual-task cost among the oldest-old may be associated with an inability to properly maintain a faster cadence while performing an arithmetic task which may be related to task prioritization.

